# Axitinib and sorafenib are potent in tyrosine kinase inhibitor resistant chronic myeloid leukemia cells

**DOI:** 10.1186/s12964-016-0129-y

**Published:** 2016-02-24

**Authors:** Sebastian Halbach, Zehan Hu, Christine Gretzmeier, Julia Ellermann, Franziska U. Wöhrle, Jörn Dengjel, Tilman Brummer

**Affiliations:** Institute of Molecular Medicine and Cell Research (IMMZ), Faculty of Medicine, University of Freiburg, Freiburg, Germany; Faculty of Biology, University of Freiburg, Freiburg, Germany; Spemann Graduate School of Biology and Medicine, University of Freiburg, Freiburg, Germany; Freiburg Institute for Advanced Studies (FRIAS), and Center for Biological Systems Analysis (ZBSA), University of Freiburg, Freiburg, Germany; BIOSS Centre for Biological Signalling Studies, University of Freiburg, Freiburg, Germany; Department of Dermatology, Medical Center, University of Freiburg, Freiburg, Germany; Deutsches Konsortium für Translationale Krebsforschung (DKTK) and Comprehensive Cancer Center Freiburg, University Medical Center, Freiburg, Germany

**Keywords:** Chronic myeloid leukemia, CML, TKI resistance, Imatinib, Sorafenib, Axitinib, Ponatinib, Gab2, Hyperactive Lyn, Bcr-Abl

## Abstract

**Background:**

Chronic myeloid leukemia (CML) is driven by the fusion kinase Bcr-Abl. Bcr-Abl tyrosine kinase inhibitors (TKIs), such as imatinib mesylate (IM), revolutionized CML therapy. Nevertheless, about 20 % of CMLs display primary or acquired TKI resistance. TKI resistance can be either caused by mutations within the Bcr-Abl kinase domain or by aberrant signaling by its effectors, e.g. Lyn or Gab2. Bcr-Abl mutations are frequently observed in TKI resistance and can only in some cases be overcome by second line TKIs. In addition, we have previously shown that the formation of Gab2 complexes can be regulated by Bcr-Abl and that Gab2 signaling counteracts the efficacy of four distinct Bcr-Abl inhibitors. Therefore, TKI resistance still represents a challenge for disease management and alternative therapies are urgently needed.

**Findings:**

Using different CML cell lines and models, we identified the clinically approved TKIs sorafenib (SF) and axitinib (AX) as drugs overcoming the resistance mediated by the Bcr Abl^T315I^ mutant as well as the one mediated by Gab2 and Lyn^Y508F^. In addition, we demonstrated that AX mainly affects the Bcr-Abl/Grb2/Gab2 axis, whereas SF seems to act independently of the fusion kinase and most likely by blocking signaling pathways up- and downstream of Gab2.

**Conclusion:**

We demonstrate that SF and AX show potency in various and mechanistically distinct scenarios of TKI resistance, including Bcr-Abl^T315I^ as well as Lyn- and Gab2-mediated resistances. Our data invites for further evaluation und consideration of these inhibitors in the treatment of TKI resistant CML.

**Electronic supplementary material:**

The online version of this article (doi:10.1186/s12964-016-0129-y) contains supplementary material, which is available to authorized users.

## Findings

**Fig. 1:**
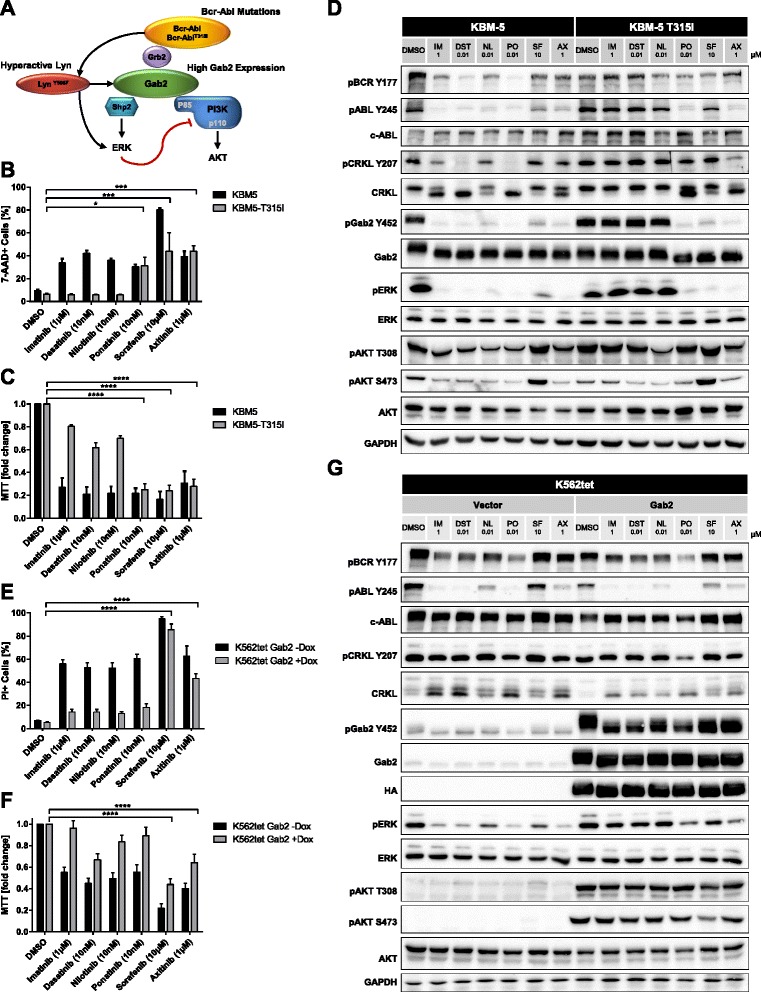
Sorafenib and axitinib can overcome TKI resistance. **a** Overview of resistance mechanisms in CML. **b**/**c** KBM5 and KBM5-T315I cells were exposed to the indicated inhibitors or DMSO for 48 h. Cells were stained with 7-AAD and assessed for viability (**b**) or metabolic activity (MTT assay) (**c**). **d** KBM5 and KBM5-T315I cells were exposed to the indicated inhibitors or DMSO for 4 h. Cells were analyzed by western blotting using the indicated antibodies. **e**/**f** K562tet Gab2 cells, exposed to 1 μg/ml doxycycline 48 h prior to the treatment or non-induced cells were treated with the indicated inhibitors or DMSO for 72 h. Cells were stained with PI and assessed for viability (**e**) or metabolic activity (MTT-assay) (**f**). **g** K562tet Vector and Gab2 cells were exposed to 1 μg/ml doxycycline 48 h prior to the treatment with the indicated inhibitors or DMSO for 4 h. Cells were analyzed by western blotting using the indicated antibodies. Relevant statistically significant effects are indicated by asterisks, all statistical data can be found in the supplement (**b**/**c**/**e**/**f**; Additional file 8 )

Chronic myeloid leukemia (CML) represents about 20 % of all cases of adult leukemia and is caused by a chromosomal translocation between chromosomes 9 and 22 leading to the expression of the fusion kinase Bcr-Abl [1]. This oncogenic tyrosine kinase generates its own signaling network with various components such as the Src kinase Lyn or the docking protein Gab2. Bcr-Abl tyrosine kinase inhibitors (TKIs), such as Imatinib mesylate (IM), revolutionized CML therapy. Nevertheless, about 20 % of CMLs display primary or acquired TKI resistance, which represents a challenge for disease management [2]. TKI resistance is often, but not exclusively, caused by mutations within the kinase domain of Bcr-Abl [3] (Fig. 1a) and can in some cases be overcome by second generation TKIs, like dasatinib (DST), nilotinib (NL), or ponatinib (PO). In the case of the gatekeeper mutation T315I, PO is the only clinically approved inhibitor showing a therapeutic effect. However, cardio-vascular side-effects often accompany PO treatment [4] and therefore alternative therapies are urgently needed. In addition, about 40 % of resistances are Bcr-Abl mutation-independent [5] and still ill-defined at the molecular level. These resistances are often caused by aberrant signaling of Bcr-Abl effectors such as the docking protein Gab2 [6–8] or the Src kinase Lyn [9] (Fig. 1a).

Using different CML cell lines and models, we aimed to identify new approaches to overcome TKI resistance caused by Bcr-Abl mutations or aberrant downstream signaling. Therefore, we screened inhibitors for their ability to inhibit the activity of T315I mutated Bcr-Abl, or to break Gab2- or Lyn-mediated resistance. Our previous work on Gab2 mediated TKI resistance in CML cells suggested that this docking protein, due to its position downstream of both growth factor receptors and Bcr-Abl [7], protects against TKIs as it can be tyrosine phosphorylated by the former and thereby drive the activation of pro-leukemogenic pathways in the absence of Bcr-Abl activity. Therefore, we chose inhibitors of growth factor receptors, as they are known to play a role in primary TKI resistance and to promote Gab2 and Lyn signaling [10, 11]. We identified the clinically approved multikinase inhibitors sorafenib (SF) and axitinib (AX) as compounds reducing the viability and metabolic activity of Bcr-Abl transformed Ba/F3 cells (Additional file 1: Figure S1A/B). While SF mainly targets Raf-1 and B-Raf, both compounds inhibit the VEGF receptors 1–3 [12, 13]. Interestingly, these inhibitors were significantly less active in non-transformed Ba/F3 cells, suggesting an inhibition specific for Bcr-Abl or its signaling network (Additional file 1: Figure S1A/B). In contrast, the FLT3/PDGFR inhibitors tandutinib (TD) and sunitinib (Sun) [14, 15] displayed activity in Bcr-Abl transformed and non-transformed Ba/F3 cells (Additional file 1: Figure S1A). Next, we tested SF and AX in KBM-5 CML cells, either expressing wildtype (wt) or mutant Bcr-Abl (T315I) from its endogenous Philadelphia chromosome [16]. Based on previous publications showing titrations of SF in IM resistant CML models [17, 18], we chose a concentration of 10 μM. For AX, we titrated the optimal inhibitor concentration using an MTT assay (Additional file 1: Figure S1C). Both inhibitors overcame TKI resistance imposed by Bcr-Abl^T315I^ (Fig. 1b/c and Additional file 1: Figure S1C). The efficacy of SF and AX was comparable to PO, while the classical TKIs IM, DST and NL only affected KBM5 cells lacking the T315I mutation (Fig. 1b/c). Interestingly, AX showed a higher efficacy in downregulating the phosphorylation of ABL (Y245) and CRKL (Y207) in Bcr-Abl^wt^ and Bcr-Abl^T315I^ cells compared to SF indicating that AX has a higher impact on the Bcr-Abl activity (Figs. 1d and Additional file 2: Figure S2A). However, SF, AX and PO reduced the phosphorylation of ERK and Gab2 (Y452) in Bcr-Abl^wt^ and Bcr-Abl^T315I^ expressing cells, whereas IM, DST and NL were only active in the former. Thus, SF and AX break T315I mediated resistance (Fig. 1d and Additional file 2: Figure S2A). In addition, these results suggest that the phosphorylation of Gab2 (Y452) might serve as a valuable biomarker in CML management. Our data is in line with very recent manuscripts by Pemovska et al. and Okabe et al. demonstrating the potency of axitinib in Bcr-Abl^T315I^ positive Ba/F3 and patient-derived cells [19, 20]. In contrast to the other inhibitors, SF provoked an upregulation of pAKT (Fig. 1d and Additional file 2: Figure S2A), which might be explained by the strong inhibition of the ERK pathway by SF and therefore the loss of a negative feedback on the PI3K/AKT pathway [21]. However, SF effectively kills Bcr-Abl^wt^ and Bcr-Abl^T315I^ expressing cells (Fig. 1b/c), which is also supported by an independent study showing effects of SF on Bcr-Abl positive cells [17].

Bcr-Abl mutation independent TKI resistance represents an underestimated and mechanistically less-defined problem in CML therapy and accounts for about 40 % of TKI refractory disease, thereby representing a serious clinical problem [5]. Therefore, we tested all clinically used TKIs of our panel, i.e. IM, DST, NL and PO but also SF and AX, in Lyn- and Gab2-mediated TKI resistance. First, we analyzed K562 cells overexpressing the hyperactive Lyn mutant Y508F, which displayed IM resistance in an independent study [9]. Again, SF and AX reduced the viability and metabolic activity in this setting (Additional file 1: Figure S1D/E). Interestingly, DST, NL and PO also overcame Lyn^Y508F^-mediated resistance (Additional file 1: Figure S1D/E), suggesting that Lyn-mediated resilience represents only a minor and less critical mechanism of TKI resistance.

Recently, we demonstrated that Gab2, a critical effector of Bcr-Abl in myeloid transformation [6], protects CML cells from IM, DST and NL [7]. We also provided several lines of evidence that this docking protein is increasingly expressed in myeloid cells from patients with TKI-refractory disease [7] or blast crisis [22], a stage known for its insensitivity to Bcr-Abl inhibitors. Therefore, we tested SF and AX in K562 cells with conditional Gab2 overexpression [7, 8]. As observed previously [7], overexpression of Gab2 conferred resistance towards IM, DST and NL (Fig. 1e/f). Interestingly, even PO failed to overcome Gab2 mediated resistance, further underscoring the critical role of Gab2 as a mediator of TKI resistance. Instead, SF and AX bypassed the protective effect of Gab2 (Fig. 1e/f). Like in KBM-5 cells, SF had a less pronounced effect on Bcr-Abl auto-phosphorylation, while AX reduced Bcr-ABL phosphorylation (Y245) (Fig. 1g and Additional file 2: Figure S2B). Interestingly, Gab2 overexpression induced an upregulation of AKT and ERK phosphorylation but not any changes in Bcr-Abl auto-phosphorylation, suggesting that Gab2 mediated resistance is caused by the activation or maintenance of the PI3K and MAPK pathways rather than by increasing Bcr-Abl activity (Fig. 1g).

**Fig. 2:**
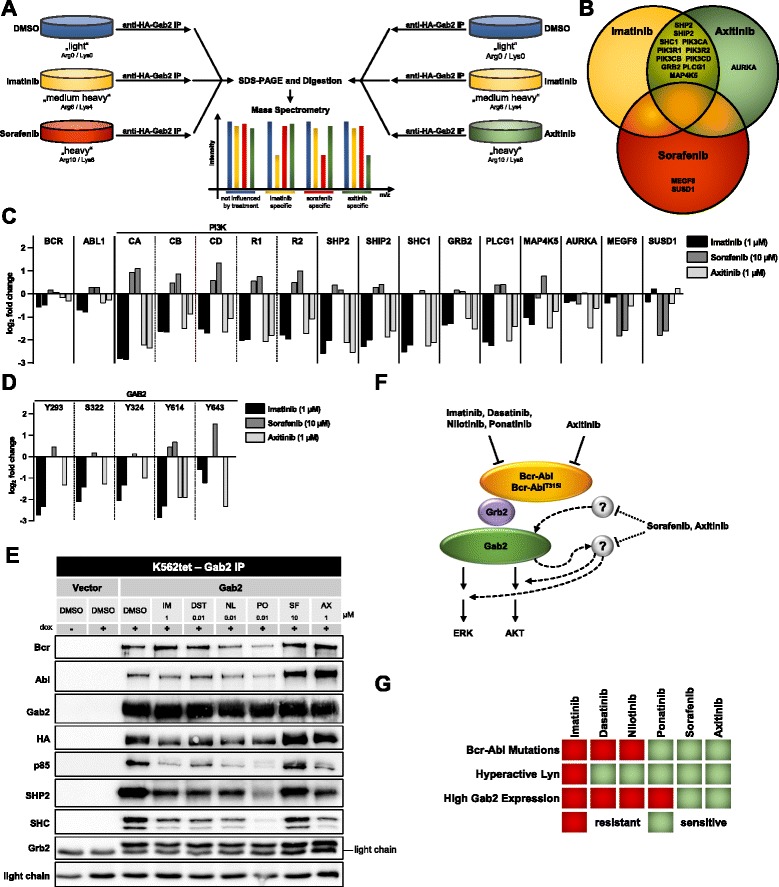
The interactome and phosphorylation status of Gab2 is differentially affected by sorafenib and axitinib. **a** Differentially SILAC labeled K562tet/Gab2-HA cells were exposed to 1 μg/ml doxycycline (to induce Gab2-HA expression) prior to treatment with either 1 μM imatinib, 10 μM sorafenib or 1 μM axitinib, and DMSO as control, respectively for 4 h. Purified Gab2 protein complexes were combined 1:1:1 and analyzed by LC-MS/MS. A biological replicate with reversed labels was performed and results of replicates correlated well. Protein interactions dependent on inhibitor sensitive phosphorylation sites will be reduced. **b** Venn diagram of imatinib, sorafenib and axitinib treatment showing TKI-sensitive Gab2 interactors. **c**/**d** TKI-sensitive changes in the Gab2 interactome (**c**) and the phosphorylation of Gab2 (**d**). Each bar represents an independent experiment (**e**) K562tet Vector and Gab2 cells were exposed to 1 μg/ml doxycycline prior to the treatment with the indicated inhibitors. Purified Gab2 complexes were analyzed using the indicated antibodies. **f** Schematic model of TKI action on the Bcr-Abl/Grb2/Gab2 signaling complex. Axitinib acts like imatinib, dasatinb, nilotinib and ponatinib mainly through the Bcr-Abl/Grb2/Gab2 axis, whereas sorafenib seems to act independently and most likely by affecting signaling pathways up- and downstream of Gab2. Due to the effects of axitinib on Gab2 mediated resistance, axitinib might act additionally also on other kinases, similar to sorafenib. **g** Diagram showing the potency of sorafenib and axitinib in all tested TKI resistances

To further investigate the influence of SF and AX on the Bcr-Abl/Gab2 signaling axis, we performed SILAC-based quantitative mass spectrometry (MS) as described previously [8]. Following labelling, HA-tagged Gab2 complexes were purified from IM, SF and AX treated K562 cells (Fig. 2a). This revealed that IM and AX remove an overlapping spectrum of Gab2 interactors (Fig. 2b/c and Additional file 3: Figure S3; Additional files 4, 5 and 6: Tables S1/S2/S3). These interactors were mostly known Gab2 partners, like PI3K components, SHP2, SHIP2, SHC, Grb2 or PLCγ but also novel interaction partners like MAP4K5 and Aurora A. These results are in line with the changes in Gab2 complex composition of IM or DS treated K562 cells [8, 23], Interestingly, SF hardly affected Gab2 interactions, but influenced proteins previously not linked to Gab2 such as MEGF8 or SUSD1. It remains to be tested whether the interaction of Gab2 to MEGF8 or SUSD1 plays a role in CML. MEGF8 and SUSD1 are single pass transmembrane proteins. MEGF8 is known to play a role in development and germline mutations of *MEGF8* have been recently linked to Carpenter syndrome subtype 2 associated with defective lateralization [24, 25], whereas SUSD1 with its two Sushi domains represents an almost uncharacterized protein. These interactions invite for further functional studies. However, the contrasting recruitment patterns of the Gab2 interaction partners illustrate the different mode of action of SF and the other TKIs used in this (Fig. 2c) and previous experiments [8].

We also analyzed the phosphorylation of Gab2 (Fig. 2d; Additional file 7: Table S4). In full agreement with the interactome data, Gab2 phosphorylation sites were markedly reduced upon IM and AX but not by SF treatment. In addition, an independent Gab2 IP was performed to confirm our MS results and to test the other inhibitors DST, NL and PO (Fig. 2e). As in the MS experiments, SF hardly influenced protein-protein interactions of Gab2, while AX downregulated the its interaction with the PI3K subunit p85, SHP2 and SHC. DST and NL had similar effects as IM. The effects of PO were in most cases more pronounced as for IM, DST and NL, suggesting a stronger inhibition of Bcr-Abl activity. Thus, like IM, DST, NL and PO, AX acts mainly on the Bcr-Abl-Grb2-Gab2 axis, whereas SF seems to act independently and most likely by affecting signaling pathways up- and downstream of Gab2. However, as AX is able to break Gab2 mediated resistance, this compound might additionally inhibit other kinases phosphorylating the docking sites on Gab2 and might therefore also cause similar effects as sorafenib (Fig. 2f). Thus, the efficacy of AX in Bcr-Abl^T315I^ mutant CML might be explained by its on-target action as a selective inhibitor for this gatekeeper mutant [19] and by “off-target” effects eliminating back-up pathways leading to Gab2 tyrosine phosphorylation and downstream signaling.

In summary, we demonstrate that SF and AX show potency in various and mechanistically distinct scenarios of TKI resistance, including Bcr-Abl^T315I^ as well as Lyn-mediated resistance. In the light of the clinically observed side effects of the currently in TKI resistant CML used inhibitor PO, SF and AX might serve as valuable alternatives. In addition, we could show that SF and AX are able to bypass the protective effect of Gab2, while PO failed to do so (Fig. 2g) as we had reported previously for other ATP competitive and allosteric inhibitors specifically designed to block Bcr-Abl activity. Our data invites for further evaluation und consideration of SF and AX in the treatment of TKI resistant CML.

### Availability of supporting data

Material, methods and supplementary statistics (Additional file 8: Supplementary Methods and Supplementary Statistics), supplementary figures (Additional files 1, 2 and 3) and data sets (Additional files 4, 55 and 6) supporting the results of this article are included within the article and its additional files.

## Additional files

Additional file 1: Figure S1. (A/B) Ba/F3 vector cells and cells transformed with pBABE Bcr-Abl were exposed to the indicated inhibitors or DMSO for 48 h. Cells were stained with 7-AAD and assessed for viability (A) or metabolic activity (MTT assay) (B). (C) KBM5 and KBM5-T315I cells were exposed to the indicated inhibitors or DMSO for 48 h. Cells were assessed for metabolic activity (MTT assay) (D/E) K562 cells overexpressing Lyn or hyperactive Lyn Y508F were exposed to the indicated inhibitors or DMSO for 48 h. Cells were stained with 7-AAD and assessed for viability (D) or metabolic activity (MTT assay) (E). Relevant statistically significant effects are indicated by asterisks, all statistical data can be found above. (PDF 176 kb) Additional file 2: Figure S2. (A/B) Western Blot quantification of Fig. 1d and 1G, *n* = 3, using FusionCapt 7.06 (Vilber Lourmat, Germany). (PDF 184 kb) Additional file 3: Figure S3. Correlation of biological replicates. GAB2 protein complexes were enriched by IP. Contaminating proteins were removed and ratios normalized to GAB2. Proteins changing significantly interactions with GAB2 by indicated inhibitor treatments are highlighted red. Signigicant affeceted proteins are determined using Significance A (MaxQuant, *p* < 0.05, BH corrected). Proteins are highlighted if minimally one IP exhibited a signifcant regulation and the other IP showed the same trend (see Additional files 4, 5 and 6: Tables S1/S2/S3). (PDF 203 kb) Additional file 4: Table S1. Anti-GAB2 IP of Imatinib vs DMSO treated cells. Experiment 1 and 2 are averages of two replicates each. SILAC ratios are normalized to GAB2 and log2 transformed. Significant affected proteins are determined using Significance A (MaxQuant, *p* < 0.05, BH corrected). Proteins are shortlisted if minimally one IP exhibited a significant regulation and the other IP showed the same trend (see Additional file 2: Figure S2). PEP: posterior error probability. (XLSX 231 kb) Additional file 5: Table S2. Anti-GAB2 IP of Sorafenib vs DMSO treated cells. SILAC ratios are normalized to GAB2 and log2 transformed. Significant affected proteins are determined using Significance A (MaxQuant, *p* < 0.05, BH corrected). Proteins are shortlisted if minimally one IP exhibited a significant regulation and the other IP showed the same trend (see Additional file 2: Figure S2). PEP: posterior error probability. (XLSX 174 kb) Additional file 6: Table S3. Anti-GAB2 IP of Axitinib vs DMSO treated cells. SILAC ratios are normalized to GAB2 and log2 transformed. Significant affected proteins are determined using Significance A (MaxQuant, *p* < 0.05, BH corrected). Proteins are shortlisted if minimally one IP exhibited a significant regulation and the other IP showed the same trend (see Additional file 2: Figure S2). PEP: posterior error probability. (XLSX 185 kb) Additional file 7: Table S4. Anti-GAB2 IP of Imatinib, Axitinib and Sorafenib vs DMSO treated cells. SILAC ratios are normalized to GAB2. Only sites with localization probabilities greater 0.75 and Andromeda scores greater 40 are shown. PEP: posterior error probability. (XLSX 12 kb) Additional file 8:
**Supplementary Methods and Supplementary Statistics.** (PDF 297 kb)

## Abbreviations

AX: Axitinib
BC: Blast crisis
CML: Chronic myeloid leukaemia
DST: Dasatinib
HA: Haemagglutinin
IM: Imatinib
MS: Mass spectrometry
NL: Nilotinib
PO: Ponatinib
SF: Sorafenib
SILAC: Stable isotope labelling by amino acids in cell culture
SUN: Sunitinib
TAN: Tandutinib
TKI: Tyrosine kinase inhibitor
