# Supplementary figures and images for: Axitinib and sorafenib are potent in tyrosine kinase inhibitor resistant chronic myeloid leukemia cells
Source: Cell Commun Signal. 2016 Feb 24;14:6. doi: 10.1186/s12964-016-0129-y (PMC4765141; doi:10.1186/s12964-016-0129-y)

**A**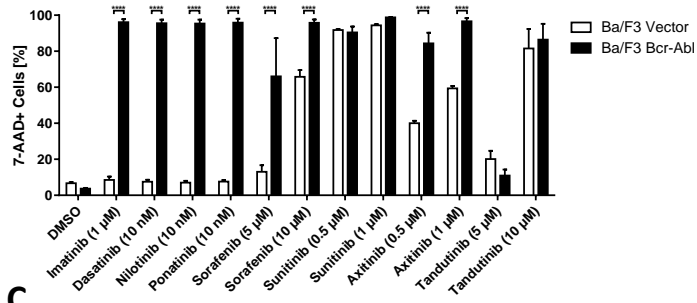**B**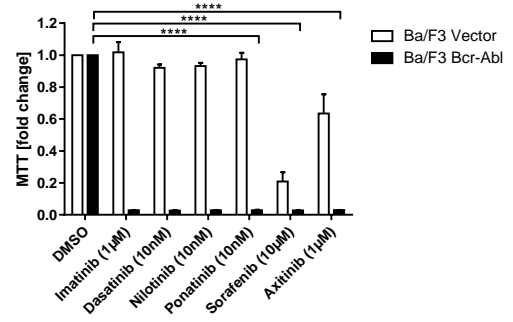**C**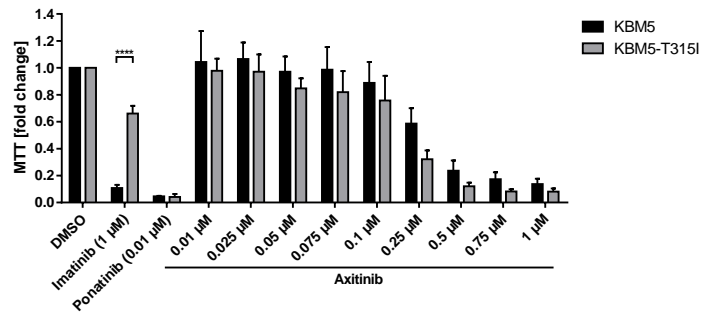**D**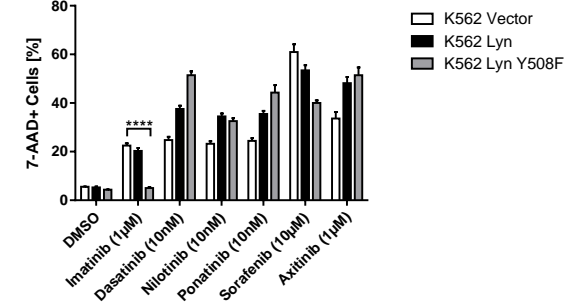**E**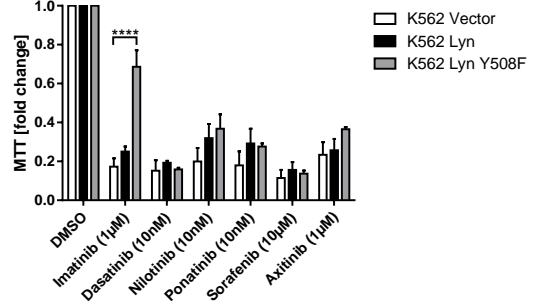

Supplement: Additional file 1: Figure S1. — (A/B) Ba/F3 vector cells and cells transformed with pBABE Bcr-Abl were exposed to the indicated inhibitors or DMSO for 48 h. Cells were stained with 7-AAD and assessed for viability (A) or metabolic activity (MTT assay) (B). (C) KBM5 and KBM5-T315I cells were exposed to the indicated inhibitors or DMSO for 48 h. Cells were assessed for metabolic activity (MTT assay) (D/E) K562 cells overexpressing Lyn or hyperactive Lyn Y508F were exposed to the indicated inhibitors or DMSO for 48 h. Cells were stained with 7-AAD and assessed for viability (D) or metabolic activity (MTT assay) (E). Relevant statistically significant effects are indicated by asterisks, all statistical data can be found above. (PDF 176 kb) [file 12964_2016_129_MOESM1_ESM.pdf]

**A**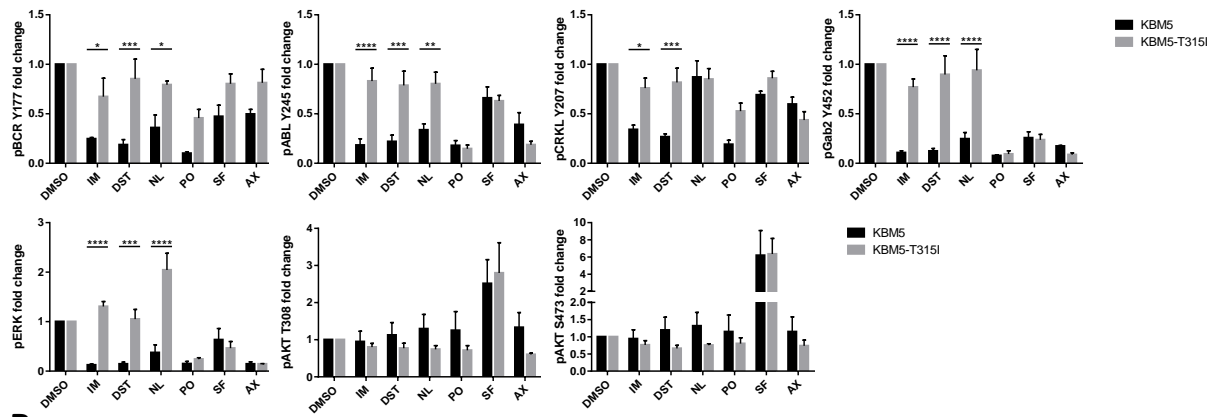**B**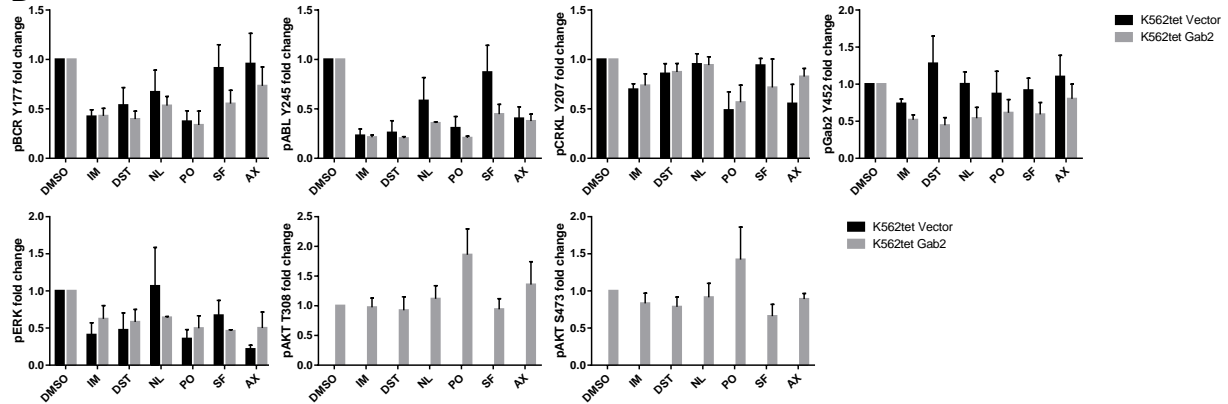

Supplement: Additional file 2: Figure S2. — (A/B) Western Blot quantification of Fig. 1d and 1G, n = 3, using FusionCapt 7.06 (Vilber Lourmat, Germany). (PDF 184 kb) [file 12964_2016_129_MOESM2_ESM.pdf]

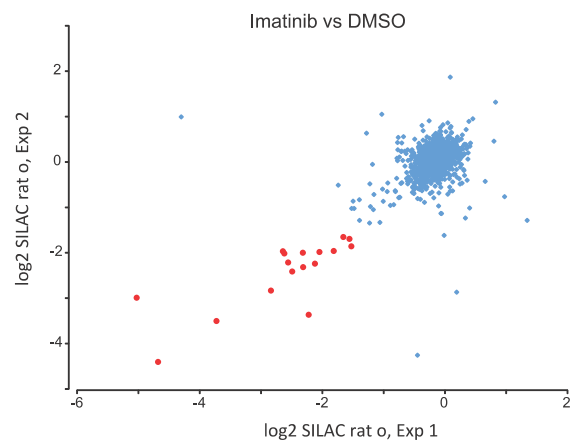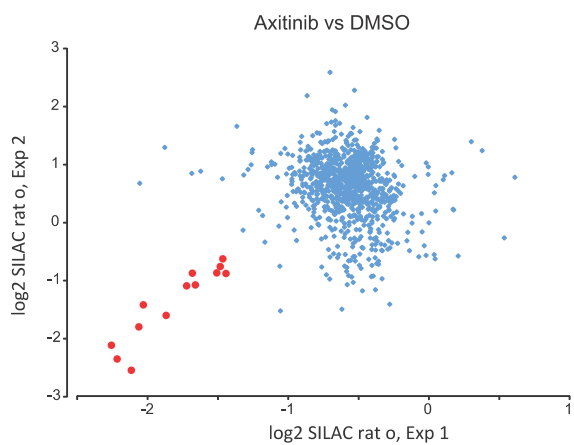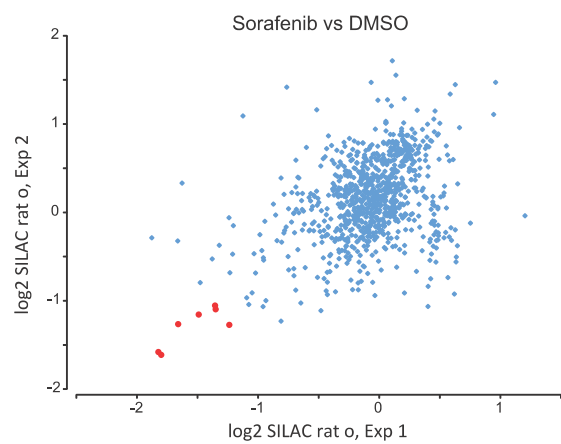

Supplement: Additional file 3: Figure S3. — Correlation of biological replicates. GAB2 protein complexes were enriched by IP. Contaminating proteins were removed and ratios normalized to GAB2. Proteins changing significantly interactions with GAB2 by indicated inhibitor treatments are highlighted red. Signigicant affeceted proteins are determined using Significance A (MaxQuant, p < 0.05, BH corrected). Proteins are highlighted if minimally one IP exhibited a signifcant regulation and the other IP showed the same trend (see Additional files 4, 5 and 6: Tables S1/S2/S3). (PDF 203 kb) [file 12964_2016_129_MOESM3_ESM.pdf]
