# Supplementary material for: Axitinib and sorafenib are potent in tyrosine kinase inhibitor resistant chronic myeloid leukemia cells
Source: Cell Commun Signal. 2016 Feb 24;14:6. doi: 10.1186/s12964-016-0129-y (PMC4765141; doi:10.1186/s12964-016-0129-y)
Supplement: Additional file 8: — Supplementary Methods and Supplementary Statistics. (PDF 297 kb) [file 12964_2016_129_MOESM8_ESM.pdf]

Supplementary Information

Axitinib and Sorafenib are potent in Tyrosine Kinase Inhibitor resistant Chronic Myeloid Leukemia Cells

Material and Methods

Cell lines

All cell lines were cultivated under water vapor saturated atmosphere at 37°C and 5% CO_2. KBM-5 cells and the sub clone line KBM-5 STI (T315I) were kindly provided by Michael Andreeff and Miloslav Beran, Houston (USA). KBM-5 is a cell line derived from a CML patient, containing multiple copies of the Philadelphia chromosome while lacking the normal ABL gene (1). KBM-5 STI (T315I) cell were derived by Ricci et al., by chronic exposure of KBM-5 cells to imatinib (2). KBM-5/KBM-5 STI (T315I) cells were maintained in IMDM (PAN-Biotech) supplemented with 10% FCS (Biochrom), 2 mM L-Glutamine (PAN-Biotech) and 10 mM HEPES (PAN-Biotech). KBM-5 STI cells were cultivated in the presence of 1 µM imatinib. The cells were switched to medium without imatinib 24 h prior to experiments. K562 and Ba/F3 cells were kind gifts from Professor Mark Guthridge, Melbourne (Australia) and Professor Michael Huber, Aachen (Germany), respectively. K562 and Ba/F3 cells were maintained in RPMI-1640 (PAN-Biotech) supplemented with 10% FCS (Biochrom), 2 mM L-Glutamine (PAN-Biotech), 10 mM HEPES (PAN-Biotech) and 50 µM beta-mercaptoethanol. Ba/F3 wt cells were cultivated in the presence of IL-3 (5% vol. conditioned medium of the X63Ag IL-3 cell line) (3).

Expression Vectors

The retroviral pBabe/Bcr-Abl-Hygro vector was derived from pMIG/p210$^{Bcr\text{-}Abl}$, a kind gift from Dr. Sebastian Herzog, Biocenter, Innsbruck Medical University (Austria). The Lyn cDNA was sub cloned from pDONR Lyn into the retroviral vector pWZL. The Lyn Y508F mutant was generated using site directed mutagenesis. The expression vectors for dox inducible expression of Gab2 in K562tet cells have been described in detail elsewhere (4, 5). Detailed cloning procedures, as well as plasmid sequences, are available upon request.

Transfection and infection of cell lines

Plat-E cells were cultured as described previously (3, 6) and transfected using polyethylenimine (Polysciences). Ba/F3 and K562 cells were infected with Plat-E culture supernatant as described previously (7). For the generation of K562/tet cells and their derivatives, 2 x 10^7 cells were electroporated with 30 µg *Fsp*I-linearized plasmid DNA. Selection of Ba/F3 and K562 cells was commenced 24 h post infection/electroporation (hygromycin B: 0.5 mg/ml, Calbiochem; blasticidin S: 5 µg/ml, Roth; puromycin: 5-7 µg/ml, Sigma).

Western blotting

K562, KBM-5 and Ba/F3 cells were processed to total cellular lysates by lysing in normal lysis buffer (50 mM TRIS/HCL pH 7.4, 1% Triton-X 100, 137 mM NaCl, 1% Glycerin, 1 mM Natrium Orthovanadate, 0.1 µg/µl Aprotinin 0.01 µg/µl Leupeptin, 1 mM AEBSF) and analyzed by western blotting as described previously(7).

List of antibodies

Antibodies used in this study were: anti-c-Abl, anti-phospho-Abl Y245, anti-phospho-AKT S473 and T308, anti-p44/p42 MAP kinase, anti-phospho-p44/p42 MAP kinase T202/Y204, anti-phospho-Bcr Y177, anti-Bcr, anti-Gab2 (26B6), anti-phospho-Gab2 Y452 (C33G1), anti-CRKL, anti-phospho-CRKL and PI3K p85 all from Cell Signaling Technology, anti-HA (3F10) from Roche Molecular Bioscience, anti-SHP2, anti-SHC and Grb2 from BD Biosciences and anti-GAPDH from Abcam.

SILAC labeling of K562 cells

K562 cells expressing Gab2-HA were cultured in DMEM (Pan Biotech, Aidenbach, Germany) containing 4.5 g/l glucose, Na-Pyruvat, 3.7 g/l NaHCO3, supplemented with penicillin/streptomycin (100 U/ml, 100 µg/ml), glutamine, 10% fetal calf serum (PAA, Coelbe, Germany). Cell populations were labeled for 6 cell doublings with either "light" AA: L-arginine and L-lysine (Arg0, Lys0, Sigma Aldrich), or "medium" AA: L-arginine-$^{13}C_6$-$^{14}N_4$ (Arg6; Sigma-Aldrich, Taufkirchen, Germany) and L-lysine-$^{2}H_4$ (Lys4; Silantes, Munich, Germany), or "heavy" AA: L-arginine-$^{13}C_6$-$^{15}N_4$ (Arg10; Sigma-Aldrich) and L-lysine-$^{13}C_6$-$^{15}N_2$ (Lys8; Silantes) in SILAC-DMEM media (Thermo Fisher, Langenselbold, Germany), supplemented with L-proline, penicillin/streptomycin (100 U/ml, 100 µg/ml), glutamine, and 10% dialyzed fetal calf serum (Gibco, Paisley, UK) which was used during labeling.

MS sample preparation

SILAC labelled cells were lysed with normal lysis buffer (50 mM TRIS/HCL pH 7.4, 1% Triton-X 100, 137 mM NaCl, 1% Glycerin, 1 mM Natrium Orthovanadate, 0.1 µg/µl Aprotine, 0.01 µg/µl leupeptin, 1 mM AEBSF), cell nuclei were pelleted and supernatants were used for affinity purification. Gab2-HA complexes were enriched using anti-HA-sepharose (Roche Applied Science) in separate IPs, one IP per SILAC label. In the last washing step, sepharose beads were combined and protein complexes eluted by incubating beads for 10 min at 95°C in SDS loading buffer containing 1 mM DTT (Sigma-Aldrich). Reduced samples were alkylated using iodoacetamide (5.5 mM) (Sigma-Aldrich). Protein mixtures were separated by SDS-PAGE (4-12% Bis-Tris gradient gel, NuPAGE (Invitrogen, Karlsruhe, Germany)), gel lanes were cut into 10 slices according to their protein content, samples in-gel digested using trypsin (Promega, Mannheim, Germany), and resulting peptide mixtures were STAGE tipped.

LC-MS/MS and data processing

Samples for LC-MS/MS were fractionated by nanoscale–HPLC on either an Agilent 1200 or an Eksigent NanoLC-ultra connected online to a LTQ-Orbitrap XL (Thermo Scientific). Peptides were separated over a linear gradient from 10-30% ACN in 0.5% acetic acid with a flow rate of 250 nl/min. All full-scan acquisition was done in the FT-MS part of the mass spectrometers in the range from m/z 350-2000 with an automatic gain control target value of 10^6 and at resolution 60,000 at m/z 400. MS acquisition was done in data-dependent mode to sequentially perform MS/MS on the five most intense ions in the full scan (Top5) in the LTQ using the following parameters. AGC target value: 5,000. Ion selection thresholds: 1000 counts and a maximum fill time of 100 ms. Wide-band activation was enabled with an activation q = 0.25 applied for 30 ms at a normalized collision energy of 35%. Singly charged and ions with unassigned charge state were excluded from MS/MS. Dynamic exclusion was applied to reject ions from repeated MS/MS selection for 45 s.

All recorded LC-MS/MS raw files were processed together in MaxQuant (8) version 1.3.0.2 with default parameters using the June 2012 UniProt human database which contains 86,898 protein sequences. For databases searching parameters were mass accuracy thresholds of 0.5 (MS/MS) and 20 ppm (precursor), Trypsin/P+DP as protease, maximum three missed cleavages,

carbamidomethylation (C) as fixed modification and oxidation (M), phosphorylation (STY) and protein N-terminal acetylation as variable modifications. MaxQuant was used to filter the identifications for a FDR below 1% for peptides, sites and proteins using forward-decoy searching. Match between runs were enabled with a retention time window of 2 min.

Phosphosites with localization probabilities ≥0.75 (class I sites) were used for bioinformatics analyses.

For Imatininb treated cells, enrichment/depletion of experiment 1 and 2 was calculated using two replicate measurements for each experiment (n=4).

Reagents and inhibitors

Imatinib mesylate, Dasatinib and Sorafenib were purchased from Santa Cruz Biotechnology and Nilotinib, Ponatinib and Axitinib from Selleck. Doxycycline hyclate was obtained from Sigma Aldrich. Anti-HA Affinity Matrix was purchased from Roche.

Statistical data

All statistics were performed using two way annova with a Bonferroni's multiple comparisons test. The following list summarizes our statistics for Figures 1 (b/c/e/f) and S1 (a/b/c/d/e).

Figures:

Figure 1b

**KBM5**		**P Value**
DMSO vs. Imatinib (1µM)	*	0,0179
DMSO vs. Dasatinib (10nM)	***	0,0009
DMSO vs. Nilotinib (10nM)	**	0,0082
DMSO vs. Ponatinib (10nM)	ns	0,0591
DMSO vs. Sorafenib (10µM)	****	< 0,0001
DMSO vs. Axitinib (1µM)	**	0,0027

**KBM5-T315I**		**P Value**
DMSO vs. Imatinib (1µM)	ns	> 0,9999
DMSO vs. Dasatinib (10nM)	ns	> 0,9999
DMSO vs. Nilotinib (10nM)	ns	> 0,9999
DMSO vs. Ponatinib (10nM)	*	0,0150
DMSO vs. Sorafenib (10µM)	***	0,0001
DMSO vs. Axitinib (1µM)	***	0,0001

**KBM5 vs. KBM5-T315I**		**P Value**
DMSO	ns	> 0,9999
Imatinib (1µM)	*	0,0114
Dasatinib (10nM)	**	0,0011
Nilotinib (10nM)	**	0,0056
Ponatinib (10nM)	ns	> 0,9999
Sorafenib (10µM)	**	0,0012
Axitinib (1µM)	ns	> 0,9999

Figure 1c

**KBM5**		**P Value**
DMSO vs. Imatinib (1µM)	****	< 0,0001
DMSO vs. Dasatinib (10nM)	****	< 0,0001
DMSO vs. Nilotinib (10nM)	****	< 0,0001
DMSO vs. Ponatinib (10nM)	****	< 0,0001
DMSO vs. Sorafenib (10µM)	****	< 0,0001
DMSO vs. Axitinib (1µM)	****	< 0,0001

**KBM5-T315I**		**P Value**
DMSO vs. Imatinib (1µM)	ns	0,1094
DMSO vs. Dasatinib (10nM)	***	0,0002
DMSO vs. Nilotinib (10nM)	**	0,0037
DMSO vs. Ponatinib (10nM)	****	< 0,0001
DMSO vs. Sorafenib (10µM)	****	< 0,0001
DMSO vs. Axitinib (1µM)	****	< 0,0001

**KBM5 vs. KBM5-T315I**		**P Value**
DMSO	ns	> 0,9999
Imatinib (1µM)	****	< 0,0001
Dasatinib (10nM)	****	< 0,0001
Nilotinib (10nM)	****	< 0,0001
Ponatinib (10nM)	ns	> 0,9999
Sorafenib (10µM)	ns	> 0,9999
Axitinib (1µM)	ns	> 0,9999

Figure 1e

K562tet Gab2 -Dox		P Value
DMSO vs. Imatinib (1µM)	****	< 0,0001
DMSO vs. Dasatinib (10nM)	****	< 0,0001
DMSO vs. Nilotinib (10nM)	****	< 0,0001
DMSO vs. Ponatinib (10nM)	****	< 0,0001
DMSO vs. Sorafenib (10µM)	****	< 0,0001
DMSO vs. Axitinib (1µM)	****	< 0,0001

K562tet Gab2 +Dox		P Value
DMSO vs. Imatinib (1µM)	ns	0,6271
DMSO vs. Dasatinib (10nM)	ns	0,6412
DMSO vs. Nilotinib (10nM)	ns	0,8954
DMSO vs. Ponatinib (10nM)	ns	0,1356
DMSO vs. Sorafenib (10µM)	****	< 0,0001
DMSO vs. Axitinib (1µM)	****	< 0,0001

K562tet Gab2 -Dox vs. K562tet Gab2 +Dox		P Value
DMSO	ns	> 0,9999
Imatinib (1µM)	****	< 0,0001
Dasatinib (10nM)	****	< 0,0001
Nilotinib (10nM)	****	< 0,0001
Ponatinib (10nM)	****	< 0,0001
Sorafenib (10µM)	ns	0,6315
Axitinib (1µM)	*	0,0161

Figure 1f

K562tet Gab2 -Dox		P Value
DMSO vs. Imatinib (1µM)	****	< 0,0001
DMSO vs. Dasatinib (10nM)	****	< 0,0001
DMSO vs. Nilotinib (10nM)	****	< 0,0001
DMSO vs. Ponatinib (10nM)	****	< 0,0001
DMSO vs. Sorafenib (10µM)	****	< 0,0001
DMSO vs. Axitinib (1µM)	****	< 0,0001

K562tet Gab2 +Dox		P Value
DMSO vs. Imatinib (1µM)	ns	0,6349
DMSO vs. Dasatinib (10nM)	****	< 0,0001
DMSO vs. Nilotinib (10nM)	*	0,0396
DMSO vs. Ponatinib (10nM)	ns	0,1757
DMSO vs. Sorafenib (10µM)	****	< 0,0001
DMSO vs. Axitinib (1µM)	****	< 0,0001

K562tet Gab2 -Dox vs. K562tet Gab2 +Dox		P Value
DMSO	ns	> 0,9999
Imatinib (1µM)	****	< 0,0001
Dasatinib (10nM)	****	< 0,0001
Nilotinib (10nM)	****	< 0,0001
Ponatinib (10nM)	****	< 0,0001
Sorafenib (10µM)	****	< 0,0001
Axitinib (1µM)	****	< 0,0001

Figure S1a

Ba/F3 Vector		P Value
DMSO vs. Imatinib (1 µM)	ns	> 0,9999
DMSO vs. Dasatinib (10 nM)	ns	> 0,9999
DMSO vs. Nilotinib (10 nM)	ns	> 0,9999
DMSO vs. Ponatinib (10 nM)	ns	> 0,9999
DMSO vs. Sorafenib (5 µM)	ns	> 0,9999
DMSO vs. Sorafenib (10 µM)	****	< 0,0001
DMSO vs. Axitinib (0.5 µM)	****	< 0,0001
DMSO vs. Axitinib (1 µM)	****	< 0,0001

Ba/F3 Bcr-Abl		P Value
DMSO vs. Imatinib (1 µM)	****	< 0,0001
DMSO vs. Dasatinib (10 nM)	****	< 0,0001
DMSO vs. Nilotinib (10 nM)	****	< 0,0001
DMSO vs. Ponatinib (10 nM)	****	< 0,0001
DMSO vs. Sorafenib (5 µM)	****	< 0,0001
DMSO vs. Sorafenib (10 µM)	****	< 0,0001
DMSO vs. Axitinib (0.5 µM)	****	< 0,0001
DMSO vs. Axitinib (1 µM)	****	< 0,0001

Figure S1b

Ba/F3 Vector		P Value
DMSO vs. Imatinib (1µM)	ns	> 0,9999
DMSO vs. Dasatinib (10nM)	ns	> 0,9999
DMSO vs. Nilotinib (10nM)	ns	> 0,9999
DMSO vs. Ponatinib (10nM)	ns	> 0,9999
DMSO vs. Sorafenib (10µM)	****	< 0,0001
DMSO vs. Axitinib (1µM)	****	< 0,0001

Ba/F3 Bcr-Abl		P Value
DMSO vs. Imatinib (1µM)	****	< 0,0001
DMSO vs. Dasatinib (10nM)	****	< 0,0001
DMSO vs. Nilotinib (10nM)	****	< 0,0001
DMSO vs. Ponatinib (10nM)	****	< 0,0001
DMSO vs. Sorafenib (10µM)	****	< 0,0001
DMSO vs. Axitinib (1µM)	****	< 0,0001

Ba/F3 Vector vs. Ba/F3 Bcr-Abl		P Value
DMSO	ns	> 0,9999
Imatinib (1 µM)	****	< 0,0001
Dasatinib (10 nM)	****	< 0,0001
Nilotinib (10 nM)	****	< 0,0001
Ponatinib (10 nM)	****	< 0,0001
Sorafenib (5 µM)	****	< 0,0001
Sorafenib (10 µM)	**	0,0010
Axitinib (0.5 µM)	****	< 0,0001
Axitinib (1 µM)	****	< 0,0001

Ba/F3 Vector vs. Ba/F3 Bcr-Abl		P Value
DMSO	ns	> 0,9999
Imatinib (1µM)	****	< 0,0001
Dasatinib (10nM)	****	< 0,0001
Nilotinib (10nM)	****	< 0,0001
Ponatinib (10nM)	****	< 0,0001
Sorafenib (10µM)	ns	0,0548
Axitinib (1µM)	****	< 0,0001

Figure S1c

KBM5		P Value
DMSO vs. Imatinib (1 µM)	****	< 0,0001
DMSO vs. Ponatinib (0.01 µM)	****	< 0,0001
DMSO vs. Axitinib 0.01 µM	ns	> 0,9999
DMSO vs. Axitinib 0.025 µM	ns	> 0,9999
DMSO vs. Axitinib 0.05 µM	ns	> 0,9999
DMSO vs. Axitinib 0.075 µM	ns	> 0,9999
DMSO vs. Axitinib 0.1 µM	ns	> 0,9999
DMSO vs. Axitinib 0.25 µM	ns	0,0719
DMSO vs. Axitinib 0.5 µM	****	< 0,0001
DMSO vs. Axitinib 0.75 µM	****	< 0,0001
DMSO vs. Axitinib 1 µM	****	< 0,0001

KBM5-T315I		P Value
DMSO vs. Imatinib (1 µM)	ns	0,2657
DMSO vs. Ponatinib (0.01 µM)	****	< 0,0001
DMSO vs. Axitinib 0.01 µM	ns	> 0,9999
DMSO vs. Axitinib 0.025 µM	ns	> 0,9999
DMSO vs. Axitinib 0.05 µM	ns	> 0,9999
DMSO vs. Axitinib 0.075 µM	ns	> 0,9999
DMSO vs. Axitinib 0.1 µM	ns	> 0,9999
DMSO vs. Axitinib 0.25 µM	***	0,0003
DMSO vs. Axitinib 0.5 µM	****	< 0,0001
DMSO vs. Axitinib 0.75 µM	****	< 0,0001
DMSO vs. Axitinib 1 µM	****	< 0,0001

KBM5 vs. KBM5-T315I		P Value
DMSO	ns	> 0,9999
Imatinib (1 µM)	****	< 0,0001
Ponatinib (0.01 µM)	ns	> 0,9999
Axitinib 0.01 µM	ns	> 0,9999
Axitinib 0.025 µM	ns	> 0,9999
Axitinib 0.05 µM	ns	> 0,9999
Axitinib 0.075 µM	ns	> 0,9999
Axitinib 0.1 µM	ns	0,0507
Axitinib 0.25 µM	ns	> 0,9999
Axitinib 0.5 µM	ns	> 0,9999
Axitinib 0.75 µM	ns	> 0,9999
Axitinib 1 µM	ns	> 0,9999

Figure S1d

K562 Vector		P Value
DMSO vs. Imatinib (1µM)	****	< 0,0001
DMSO vs. Dasatinib (10nM)	****	< 0,0001
DMSO vs. Nilotinib (10nM)	****	< 0,0001
DMSO vs. Ponatinib (10nM)	****	< 0,0001
DMSO vs. Sorafenib (10µM)	****	< 0,0001
DMSO vs. Axitinib (1µM)	****	< 0,0001

Figure S1e

K562 Vector		P Value
DMSO vs. Imatinib (1µM)	****	< 0,0001
DMSO vs. Dasatinib (10nM)	****	< 0,0001
DMSO vs. Nilotinib (10nM)	****	< 0,0001
DMSO vs. Ponatinib (10nM)	****	< 0,0001
DMSO vs. Sorafenib (10µM)	****	< 0,0001
DMSO vs. Axitinib (1µM)	****	< 0,0001

K562 Lyn		P Value
DMSO vs. Imatinib (1µM)	****	< 0,0001
DMSO vs. Dasatinib (10nM)	****	< 0,0001
DMSO vs. Nilotinib (10nM)	****	< 0,0001
DMSO vs. Ponatinib (10nM)	****	< 0,0001
DMSO vs. Sorafenib (10µM)	****	< 0,0001
DMSO vs. Axitinib (1µM)	****	< 0,0001

K562 Lyn Y508F		P Value
DMSO vs. Imatinib (1µM)	ns	> 0,9999
DMSO vs. Dasatinib (10nM)	****	< 0,0001
DMSO vs. Nilotinib (10nM)	****	< 0,0001
DMSO vs. Ponatinib (10nM)	****	< 0,0001
DMSO vs. Sorafenib (10µM)	****	< 0,0001
DMSO vs. Axitinib (1µM)	****	< 0,0001

K562 Vector vs. K562 Lyn Y508F		P Value
DMSO	ns	> 0,9999
Imatinib (1µM)	****	< 0,0001
Dasatinib (10nM)	****	< 0,0001
Nilotinib (10nM)	**	0,0012
Ponatinib (10nM)	****	< 0,0001
Sorafenib (10µM)	****	< 0,0001
Axitinib (1µM)	****	< 0,0001

K562 Lyn		P Value
DMSO vs. Imatinib (1µM)	****	< 0,0001
DMSO vs. Dasatinib (10nM)	****	< 0,0001
DMSO vs. Nilotinib (10nM)	****	< 0,0001
DMSO vs. Ponatinib (10nM)	****	< 0,0001
DMSO vs. Sorafenib (10µM)	****	< 0,0001
DMSO vs. Axitinib (1µM)	****	< 0,0001

K562 Lyn Y508F		P Value
DMSO vs. Imatinib (1µM)	***	0,0003
DMSO vs. Dasatinib (10nM)	****	< 0,0001
DMSO vs. Nilotinib (10nM)	****	< 0,0001
DMSO vs. Ponatinib (10nM)	****	< 0,0001
DMSO vs. Sorafenib (10µM)	****	< 0,0001
DMSO vs. Axitinib (1µM)	****	< 0,0001

K562 Vector vs. K562 Lyn Y508F		P Value
DMSO	ns	> 0,9999
Imatinib (1µM)	****	< 0,0001
Dasatinib (10nM)	ns	> 0,9999
Nilotinib (10nM)	ns	0,1494
Ponatinib (10nM)	ns	> 0,9999
Sorafenib (10µM)	ns	> 0,9999
Axitinib (1µM)	ns	0,4328

ns = not significant

Supplementary References

1. Beran M, Pisa P, O'Brien S, Kurzrock R, Siciliano M, Cork A, et al. Biological properties and growth in SCID mice of a new myelogenous leukemia cell line (KBM-5) derived from chronic myelogenous leukemia cells in the blastic phase. Cancer Res. 1993 Aug 1;53(15):3603-10.

2. Ricci C, Scappini B, Divoky V, Gatto S, Onida F, Verstovsek S, et al. Mutation in the ATP-binding pocket of the ABL kinase domain in an STI571-resistant BCR/ABL-positive cell line. Cancer Res. 2002 Nov 1;62(21):5995-8.

3. Brummer T, Larance M, Abreu MT, Lyons RJ, Timpson P, Emmerich CH, et al. Phosphorylation-dependent binding of 14-3-3 terminates signalling by the Gab2 docking protein. Embo J. 2008 Aug 7;27(17):2305-16.

4. Herr R, Wohrle FU, Danke C, Berens C, Brummer T. A novel MCF-10A line allowing conditional oncogene expression in 3D culture. Cell communication and signaling : CCS. 2011;9:17.

5. Wöhrle FU, Halbach S, Aumann K, Schwemmers S, Braun S, Auberger P, et al. Gab2 signaling in chronic myeloid leukemia cells confers resistance to multiple Bcr-Abl inhibitors. Leukemia : official journal of the Leukemia Society of America, Leukemia Research Fund, UK. 2013 Jan;27(1):118-29.

6. Morita S, Kojima T, Kitamura T. Plat-E: an efficient and stable system for transient packaging of retroviruses. Gene Ther. 2000 Jun;7(12):1063-6.

7. Brummer T, Schramek D, Hayes VM, Bennett HL, Caldon CE, Musgrove EA, et al. Increased proliferation and altered growth factor dependence of human mammary epithelial cells overexpressing the Gab2 docking protein. The Journal of biological chemistry. 2006 Jan 6;281(1):626-37.

8. Cox J, Mann M. MaxQuant enables high peptide identification rates, individualized p.p.b.-range mass accuracies and proteome-wide protein quantification. Nature biotechnology. 2008 Dec;26(12):1367-72.
